# 1,2-Benzothiazine Derivatives as Anti-HIV and Anti-HCV Agents: Structure–Activity Relationships and Research Perspectives

**DOI:** 10.3390/ijms27177800

**Published:** 2026-08-31

**Authors:** Izabela Topolska, Berenika M. Szczęśniak-Sięga

**Affiliations:** 1 Student Science Club of Medicinal Chemistry, Department of Medicinal Chemistry, Faculty of Pharmacy, Wroclaw Medical University, Borowska 211, 50-556 Wrocław, Poland; izabela.topolska@student.umw.edu.pl; 2 Department of Medicinal Chemistry, Faculty of Pharmacy, Wroclaw Medical University, Borowska 211, 50-556 Wrocław, Poland

**Keywords:** 1,2-benzothiazine, anti-HIV agents, anti-HCV agents, structure–activity relationships

## Abstract

1,2-Benzothiazine derivatives have attracted attention as structurally versatile scaffolds for antiviral drug discovery. Their sulfur-containing heterocyclic core supports diverse substitution patterns, allowing modulation of physicochemical properties and biological activity through medicinal chemistry optimization. This review summarizes published studies on 1,2-benzothiazine-based derivatives as potential inhibitors of human immunodeficiency virus (HIV) and hepatitis C virus (HCV), with particular emphasis on structure–activity relationships (SAR). Comprehensive SAR analyses indicate that substitutions at the N-1 and C-3 positions play a pivotal role in modulating antiviral potency and selectivity. Among the reported compounds, the most selective anti-HIV agent was a pyrazole-substituted 1,2-benzothiazine (**9h**) bearing a 2-amino-4-methylthiazolehydrazidoacetyl moiety, which exhibited an EC_50_ value of 3.8 μM against HIV-1 in primary human peripheral blood mononuclear cells. For HCV, the most promising derivative was a pyrazolebenzothiazine (**5b**) containing a 4-chloro substituent in the N-1 phenyl ring and a *p*-methanesulfonamidophenyl group at the C-3 position, demonstrating an IC_50_ value of 7.9 μM in the NS5B polymerase assay. Despite showing antiviral activity against both HIV and HCV, all tested compounds, including the most active derivatives, were considerably less potent than the corresponding reference drugs. Further optimization is therefore needed to improve their antiviral potency.

## 1. Introduction

Viral diseases represent a major group of infectious conditions caused by viruses that are obligate intracellular parasites capable of replicating exclusively within host cells [1]. The diversity of viruses in terms of structure and genetic material translates into a wide spectrum of clinical manifestations and disease courses, ranging from mild infections to severe and chronic conditions [2,3,4,5,6,7,8]. Infection occurs through various routes of transmission, and the host immune response plays a key role in controlling infection; however, some viruses are capable of evading host defense mechanisms [9,10,11]. Viral diseases pose a serious public health challenge due to their high infectivity, limited availability of causal treatment, and the dynamic genetic variability of viruses, which complicates the development of effective therapeutic and preventive strategies [12].

Infection with the human immunodeficiency virus (HIV) and the hepatitis C virus (HCV) continues to be a major global public health concern. By the end of 2024, approximately 40.8 million people worldwide were living with HIV, and in 2024 an estimated 1.3 million new infections and about 630 thousand AIDS-related deaths were recorded—despite progress in diagnostics and therapy [13]. Infection with the hepatitis C virus (HCV), in turn, leads to significant morbidity and mortality associated with liver diseases. It is estimated that currently around 50 million people worldwide live with chronic HCV infection, with approximately 1 million new infections occurring annually, while deaths related to disease complications (mainly liver cirrhosis and hepatocellular carcinoma) affect hundreds of thousands of people each year [14].

**Figure 1:**
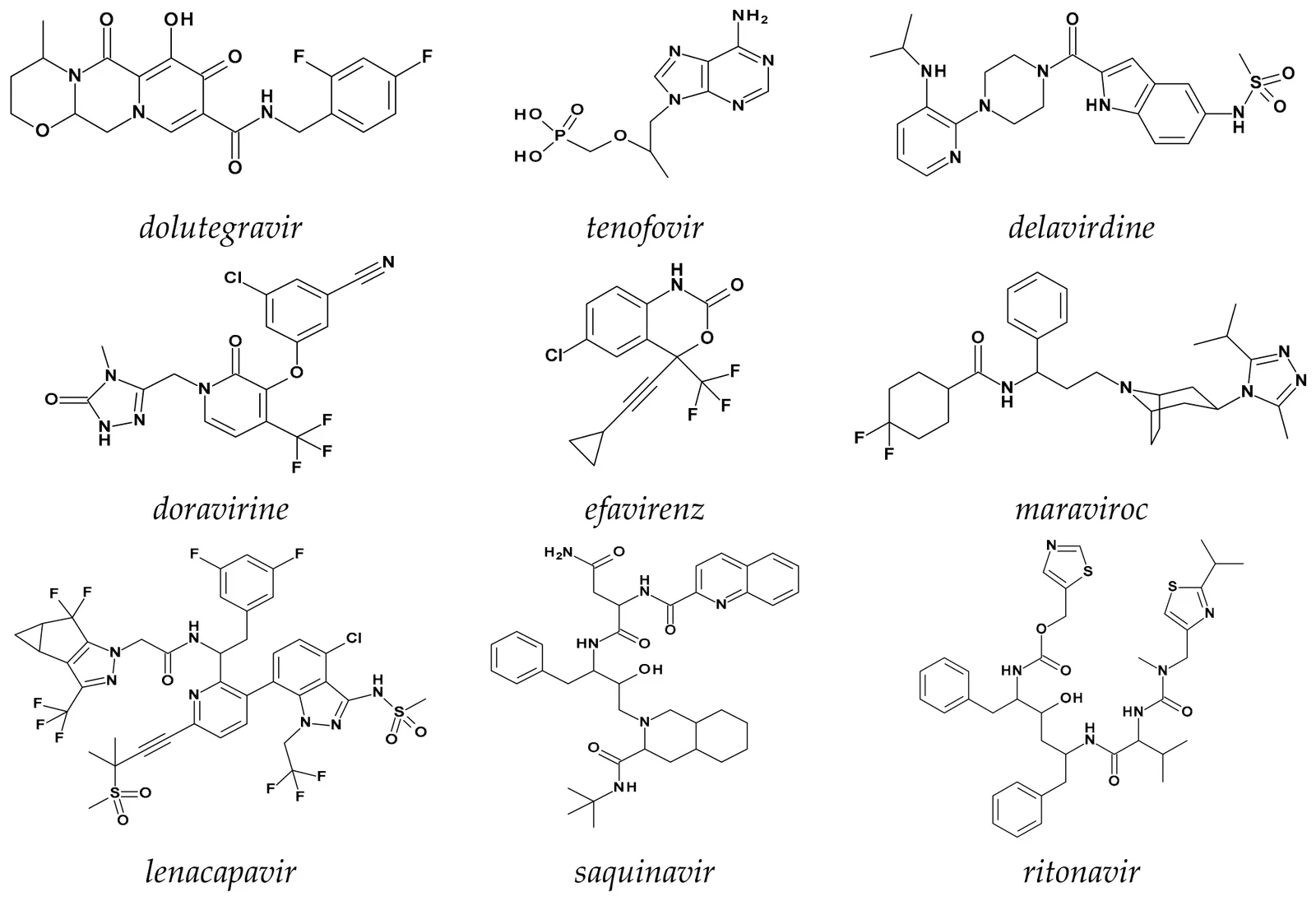
Examples of drugs used in the treatment of HIV infection.

The treatment of HIV infection is based on continuous Antiretroviral Therapy (ART), which inhibits viral replication and enables the achievement of viral suppression to an undetectable level [15,16]. Early initiation of ART significantly improves prognosis, reducing the risk of progression to AIDS, as well as the duration and severity of comorbid conditions, and also limiting virus transmission. The principle that “Undetectable = Untransmittable” (U = U) has been widely adopted [17]. Typically, combination regimens consisting of three drugs with different mechanisms of action are used, including integrase strand transfer inhibitors (INSTIs, e.g., dolutegravir, lenacapavir, raltegravir, elvitegravir), nucleoside reverse transcriptase inhibitors (NRTIs, e.g., lamivudine, zidovudine, tenofovir, emtricitabine), non-nucleoside reverse transcriptase inhibitors (NNRTIs, e.g., efavirenz, delavirdine, doravirine, etravirine, nevirapine), protease inhibitors (PIs, e.g., indinavir, ritonavir, saquinavir, atazanavir), and fusion inhibitors (FIs, e.g., enfuvirtide, maraviroc) (Figure 1) [18,19,20,21]. ART has transformed HIV infection from a nearly universally fatal condition into a chronic disease requiring long-term treatment; however, it still cannot be permanently cured. In this context, the development of novel therapeutic strategies enabling complete viral eradication remains a major challenge of modern medicine.

**Figure 2:**
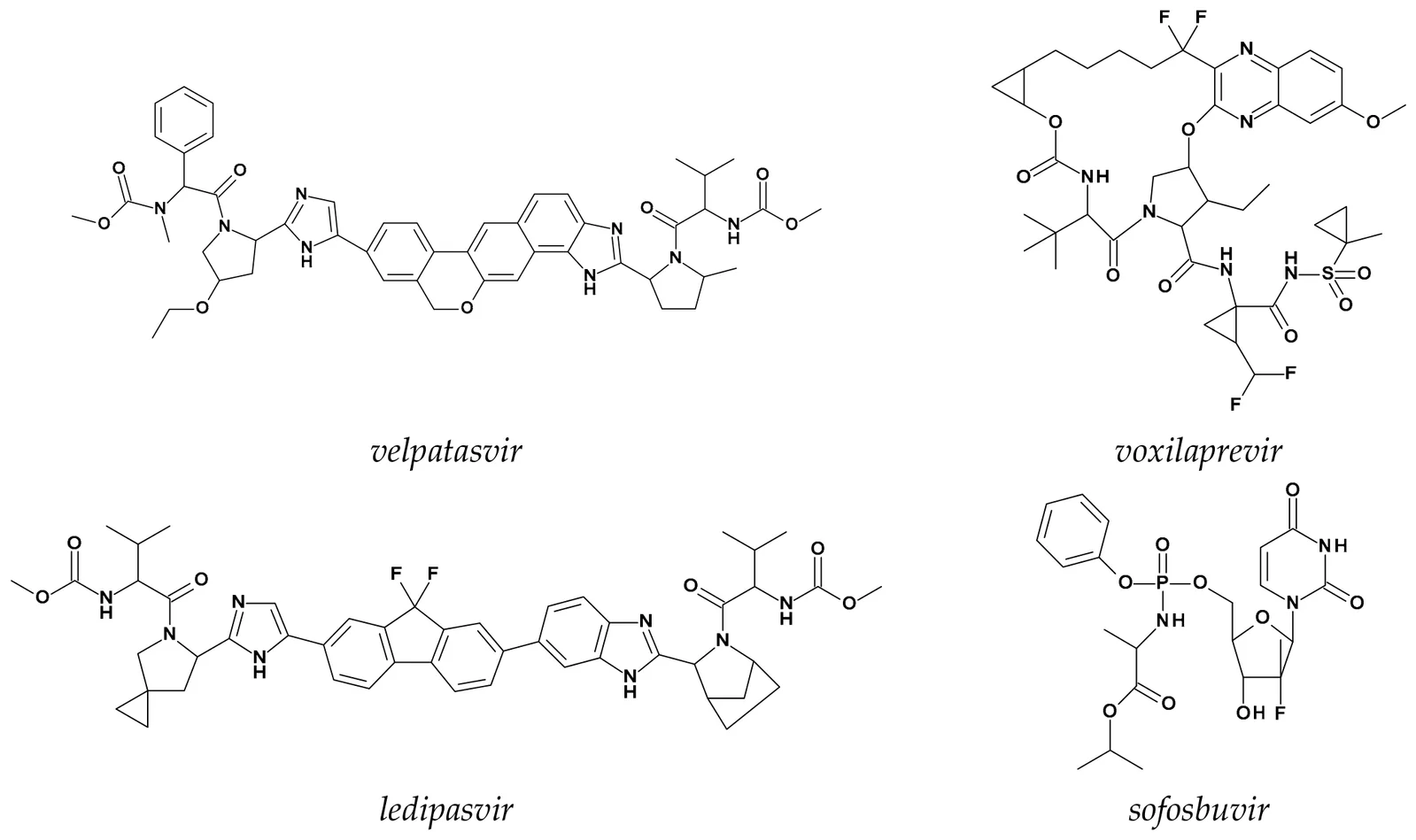
Examples of drugs used in the treatment of HCV infections.

In contrast, HCV infection therapy is considerably more effective than HIV treatment. Approximately 30% of individuals infected with HCV may spontaneously clear the virus within the first months after infection; however, in the majority of cases, the infection progresses to a chronic form characterized by persistent viremia and ongoing liver inflammation [22,23]. The current prognosis for HCV infection has improved markedly due to the introduction of direct-acting antiviral agents (DAAs), which enable cure in over 95% of treated patients after a short treatment course (usually 8–12 weeks) [24,25]. Modern pangenotypic regimens (e.g., sofosbuvir + daclatasvir, sofosbuvir + ledipasvir, sofosbuvir + velpatasvir + voxilaprevir, or glecaprevir + pibrentasvir) can eliminate the virus, thereby preventing further liver damage (Figure 2) [26,27,28]. However, following HCV exposure, most infections are asymptomatic or present with non-specific symptoms in the acute phase, which makes early diagnosis and treatment difficult. Additionally, limited diagnostic capacity and restricted access to treatment in many countries remain significant barriers to effective disease control.

Antiviral drugs used in the treatment of HIV and HCV constitute a diverse group of small-molecule organic compounds specifically designed to target molecular components of the viral replication cycle. Many of them are nucleoside and nucleotide analogs, resulting in the termination of viral DNA or RNA synthesis [29]. Other compounds possess structures that enable specific binding to viral enzymes, such as polymerases, proteases, or integrases [30]. These compounds often exhibit polycyclic structures containing various heterocyclic rings, including six-membered rings (e.g., pyridine, pyrimidine, piperazine, oxazine) and five-membered rings (e.g., thiazole, pyrazole, imidazole, pyrrolidine) (see Figure 1 and Figure 2). This structural diversity has also prompted the investigation of thiazine derivatives as potential antiviral agents. Thiazine derivatives exhibit a wide spectrum of pharmacological activities, including antibacterial properties, exemplified by cephalosporins (five generations), which are β-lactam antibiotics with a broad spectrum of bactericidal activity [31,32,33].

**Figure 3:**
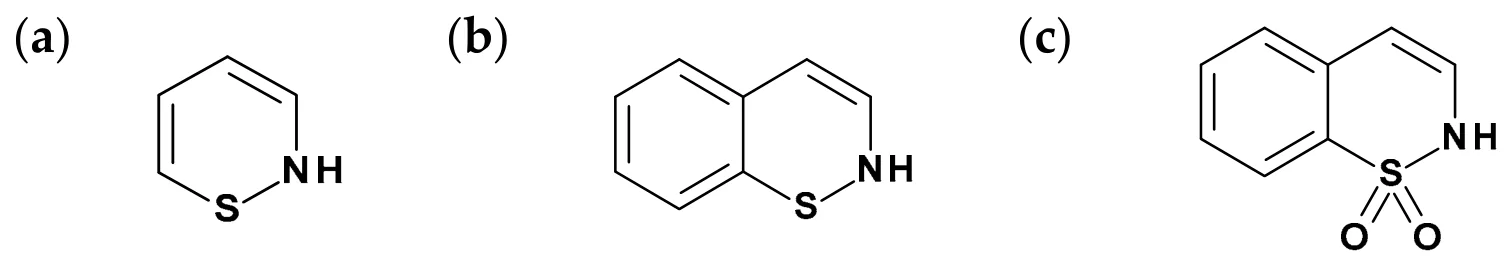
Structures of 1,2-thiazine (**a**), 1,2-benzothiazine (**b**), and 1,2-benzothiazine 1,1-dioxide (**c**).

Thiazine is a six-membered heterocyclic ring containing two heteroatoms: a sulfur atom and a nitrogen atom. Depending on the relative positions of these heteroatoms within the ring, three thiazine isomers are distinguished: 1,2-, 1,3-, and 1,4-thiazine [34,35]. Thiazines often exhibit pharmacological activity, particularly when fused with another ring, such as a benzene ring. Fusion of the 1,2-thiazine ring (Figure 3a) with a benzene ring leads to the formation of a bicyclic system known as 1,2-benzothiazine (Figure 3b). Among the many possible benzothiazine isomers, 1,2-benzothiazine derivatives with an oxidized sulfur atom (Figure 3c) exhibit particularly broad biological properties [36,37,38,39,40,41,42,43,44,45,46,47,48,49,50,51,52,53,54,55].

This review provides an overview of the antiviral properties of 1,2-benzothiazine derivatives, covering studies published between 2013 and 2021. This timeframe reflects the available literature, as no studies on the antiviral activity of this class of compounds were published between 2022 and 2026. The review summarizes the current knowledge of 1,2-benzothiazine derivatives and highlights the current limitations and challenges associated with their biological activity. These compounds have been investigated for activity against HIV and HCV; therefore, this review is divided into two sections, addressing HIV and HCV separately. As studies on anti-HCV activity were the first to be conducted in this group of compounds, they are discussed first.

## 2. 1,2-Benzothiazine Derivatives as Anti-HCV Agents

In 2013, Barreca et al. reported the synthesis and evaluation of new pyrazole derivatives of 1,2-benzothiazine as potential selective anti-HCV agents [56]. The target of these compounds was the NS5B polymerase (RNA-dependent RNA polymerase), which catalyzes viral RNA synthesis. Due to its sequence and structural differences compared to human DNA and RNA polymerases, it represents an attractive target for selective HCV inhibitors [57,58]. The study focused on the allosteric PSI site, located at the interface of the thumb and palm domains, near the active site. Several chemotypes that bind to this site have been described in the literature, including hydroxyquinoline-benzothiadiazine (BTDZ) and hydroxyquinoline-benzothiazine (BTZ) derivatives [59,60,61]. Based on these structures, a pyrazolobenzothiazine derivative (compound **1**, Table 1) was identified through virtual screening of an internal library as a starting point for the rational design of NS5B inhibitors. A synthetic route to new pyrazolobenzothiazines bearing a *p*-methanesulfonamidophenyl group at the C-3 position was developed, resulting in twelve new compounds (Table 1). The synthesized compounds were evaluated using a combination of biochemical and cell-based assays to determine their binding affinity for NS5B polymerase, their ability to inhibit the enzyme, and their anti-HCV activity [56].

In the first assay, the interaction between the synthesized compounds and HCV NS5B genotype 1b was evaluated using an SPR-based biosensor. The results were expressed as the dissociation constant (KD), which corresponds to the concentration at which half of the binding sites are occupied by the ligand. Apparent affinities were determined for all compounds except two, i.e., **2c** and **2g** (Table 1). Compound **2c** exhibited poor solubility, whereas compound **2g** showed no detectable interaction with the target. For the remaining compounds, an increase in affinity (KD values ranging from 14 to 165 μM) was observed compared to the starting compound **1** (KD > 300 μM), which was attributed to the introduction of the *p*-methanesulfonamidophenyl fragment at the C-3 position. Among these, compound **2e**, bearing an *m*-nitrophenyl substituent at the N-1 position, exhibited the highest affinity for the polymerase (Table 1).

In a subsequent study, the inhibitory activity of all synthesized compounds against NS5B RdRp was evaluated using a standard primer-dependent elongation assay with a poly rA/U12 template-primer and recombinant HCV NS5BCΔ21. All compounds were initially tested at a concentration of 50 µM to identify candidates for NS5B inhibitors showing ≥ 50% inhibition of NS5B RdRp activity. Eight compounds that met this criterion (**2a**–**d**, **2f**, **2g**, **2i**, and **2j**) were further evaluated, yielding IC_50_ values in the range of 3.9–39.9 μM. The reference compound used was hydroxyquinoline-benzothiadiazine (BTDZ), one of the best-studied HCV polymerase inhibitors [61,62]. This study confirmed the rationale for introducing the *p*-methanesulfonamidophenyl group at the C-3 position, as the starting compound **1**, which lacked this group, showed only 11% inhibition at a concentration of 50 μM. Furthermore, the position of the substituent on the N-1 phenyl ring in series 2 influenced anti-NS5B activity. Substitution at the *para* position (**2c**, **2g**, and **2j**) was more favorable than substitution at the *ortho* (**2b**, **2f**, and **2i**) and *meta* (**2a**, **2e**, and **2h**) positions. For instance, *p*-fluoro N-1-phenyl-pyrazolobenzothiazine **2c** showed the highest activity, with an IC_50_ value of 3.9 μM. Nevertheless, the tested compounds exhibited significantly lower activity than the reference compound BTDZ (IC_50_ = 0.085 μM).

In the third assay, the selective antiviral activity of the studied compounds was evaluated in an HCV genotype 1b subgenomic replicon system (Huh 5-2). From the dose–response curves, the compound concentration inhibiting 50% of viral replication (EC_50_), as well as the concentration reducing host cell viability by 50% (CC_50_), were determined. These values were used to calculate the selectivity index (SI = CC_50_/EC_50_), which is a measure of the compound’s therapeutic potential. Analysis of the data showed that the studied compounds did not exhibit significant cytotoxicity, except for derivatives **2g**, **2h**, and **2j**. Good activity, combined with the absence of cytotoxic effects, was observed among the N-1-fluorophenyl derivatives, with compound **2a** showing the highest activity (EC_50_ = 7.5 μM). Notably, it achieved 90% inhibition of HCV replication at a non-toxic concentration (EC_90_ = 42 μM). Overall, the results indicate that compound **2a** is the most promising candidate for an HCV replication inhibitor with a potentially novel mechanism of action.

Continuing their research, Manfroni et al. published the synthesis of a new series of pyrazolobenzothiazines, designed to elucidate the SAR of this class of NS5B inhibitors and to identify compounds with improved anti-HCV activity [63]. Previous studies had shown that the *p*-methanesulfonamide group and the amide bond are important for anti-HCV activity, with a fluorine atom at the *meta* position being the most favorable substituent on the N-1 phenyl ring. To enable a more detailed SAR analysis, various substituents were introduced on the N-1 phenyl ring, including chlorine, bromine, methyl, methoxy, and trifluoromethyl groups at different positions. A derivative bearing a cyclohexyl substituent was also synthesized to assess its impact on antiviral activity, along with compounds lacking the methanesulfonamide group (compound **6**), containing a carbamoylmethoxy group (compound **7**), and with a reversed amide bond (compound **8**) (Table 2).

All compounds were initially evaluated for NS5B polymerase inhibition at 50 µM to identify candidates showing ≥50% inhibition of NS5B RdRp activity. Fifteen compounds meeting this criterion (**5a**–**5i**, **5l**, **5m**, **5o**, **5p**, **5r**, and **5t**) were further screened for NS5B inhibition, yielding IC_50_ values ranging from 2.7 to 43 μM. The most active compound was **5p**, containing two chlorine atoms at the *meta* and *para* positions, with an IC_50_ of 2.7 μM. Both the type and position of the substituent on the phenyl ring significantly influenced anti-HCV activity. Compounds containing chlorine or bromine atoms at the *meta* position (**5a**, **5d**, **5l**) exhibited the highest activity, whereas compounds with methyl or trifluoromethyl groups (**5g**, **5h**, **5i**, **5l**, **5m**, **5n**) showed lower activity. Compounds bearing a methoxy group, irrespective of its position, were inactive (**5j**, **5k**), highlighting the importance of a lipophilic substituent at the N-1 position. A bulky cyclohexyl substituent (**5t**) was also unfavorable for antiviral activity. Compounds **6**, **7**, and **8** were inactive as well, further confirming the key role of the *p*-methanesulfonamide group and the amide linker in NS5B polymerase inhibition.

In the subsequent assay, all compounds were evaluated for their anti-HCV activity (EC_50_, EC_90_) using Huh-5-2 cells harboring a genotype 1b HCV subgenomic replicon (Table 2). Simultaneously, the cytotoxic effects (CC_50_) were assessed using the MTS assay, and the selectivity index (SI) was calculated. Compounds showing ≥70% inhibition of viral RNA replication without detectable cytotoxicity at the tested concentration were considered selectively active. The cell-based assays revealed that with the exception of derivatives **5n**, **5u**, and **8**, all remaining compounds were active against HCV, displaying EC_50_ values below 15 µM. However, because of either a significant toxic effect as apparent from the MTS assay or low SI values (≤10), compounds **5f**, **5o**, **5t**, and **6** were considered as false positives (EC_50_ not reliable). After dose−response curve analysis, derivatives **5c**, **5i**, **5m**, **5p**, **5r**, **5s**, and **7** were also deemed as unsuitable, as they displayed antimetabolic effect. Only derivatives **5a**, **5b**, **5d**, **5e**, **5g**, **5h**, **5k**, and **5l** demonstrated selective anti-HCV activity without any antimetabolic effects. Following microscopic analysis aimed at detecting subtle changes in cell morphology, only **5b**, **5k**, and **5h** were considered true anti-HCV agents, as they did not induce morphological changes after 72 h of treatment. The morphological alterations induced by the other pyrazolobenzothiazine derivatives might be partially attributable to drug-induced steatosis. One possible explanation is the inhibition of mitochondrial β-oxidation, although other mechanisms underlying drug-induced steatosis cannot be excluded [63].

In direct comparison, the most selective compound, **5b**, exhibited anti-HCV activity comparable to the reference compound **2a**. Unlike the parent compound, **5b** did not induce any morphological changes, highlighting it as a more promising antiviral agent. Although compounds **5a** and **5p** exhibited stronger inhibition of the NS5B polymerase than **5b**, with **5p** showing the highest enzymatic potency (IC_50_ = 2.7 µM), biochemical activity alone did not determine the overall biological potential of the compounds. The subsequent cell-based assays demonstrated that **5b** had a particularly favorable profile, combining selective anti-HCV activity with the absence of detectable cytotoxicity and noticeable morphological changes in Huh-5-2 cells.

In contrast, the higher NS5B inhibitory potency observed for **5a** and **5p** did not translate into a superior overall cellular profile. Therefore, **5b** was considered the most promising derivative based on the balance between antiviral activity, selectivity, and cellular tolerability rather than on enzymatic potency alone [63].

## 3. 1,2-Benzothiazine Derivatives as Anti-HIV Agents

In 2014, Aslam et al. reported the synthesis of new 1,2-benzothiazine derivatives designed as potential inhibitors of HIV replication [64]. The benzothiazine scaffold was fused with a pyrazole ring, and various heterocyclic substituents (e.g., thiophene, thiazole, furan, pyridine, or pyrrole), commonly found in established anti-HIV agents, were introduced into this tricyclic system [65]. The rationale for anticipating antiviral activity was based on the broad-spectrum activity of 1,2-benzothiazine derivatives and the findings of Barreca et al., who confirmed the activity of similar compounds against HCV [56]. In total, twenty-three derivatives were synthesized, each bearing a heterocyclic ring attached via an acethydrazide group to the thiazine nitrogen atom (Table 3). The synthetic procedures were optimized by performing reactions in an ultrasonic bath, which increased yields and reduced reaction times [64].

All new compounds were evaluated for their ability to inhibit HIV-1 replication in activated primary human peripheral blood mononuclear cells (PBMCs). Anti-HIV-1 activity was assessed following the method described by Schinazi [66], with results expressed as EC_50_ and EC_90_ (Table 3). Cytotoxicity of the compounds was evaluated on PBMC, CEM, and Vero cell lines, using the procedure described by Stuyver [67]. CEM cells are lymphoblastic cells originally derived from a child with acute lymphoblastic leukemia, whereas Vero cells originate from African green monkey kidney. In all assays, zidovudine (AZT), a nucleoside reverse transcriptase inhibitor (NRTI) used clinically for HIV treatment, served as the reference compound [68].

Of the twenty-three compounds tested, seven exhibited modest anti-HIV activity, with EC_50_ values below 20 μM, while the remaining compounds were inactive. Most active compounds were thiophene derivatives (**9a**, **9b**, **9e**, **9i**, **9j**, and **9m**), and one contained a thiazole ring (**9h**). Substitution of the thiophene ring with two chlorine atoms at positions 3 and 5 produced the most active compound (**9i**, EC_50_ = 3.2 μM), and a similar activity was observed for the thiazole derivative **9h** (EC_50_ = 3.8 μM). In contrast, derivatives containing furan, pyrrole, pyridine, or naphthalene rings were completely inactive (EC_50_ > 100 μM). Cytotoxicity studies revealed that **9i**, despite its high antiviral activity, was highly toxic to PBMCs, CEM, and Vero cells, excluding it from further consideration. Compound **9h**, although non-toxic (IC_50_ > 100 μM), demonstrated limited potency compared to the reference drug zidovudine (EC_50_ = 0.00037 μM), and thus does not represent a promising candidate for anti-HIV therapy.

In the same year, the authors reported a new series of 1,2-benzothiazine derivatives and evaluated their activity as HIV replication inhibitors [69]. Twenty-one compounds (**10a**–**u**) were obtained using classical heating and microwave-assisted methods (Table 4). In these derivatives, an acethydrazide group bearing a phenyl ring substituted at various positions with electron-donating (e.g., OH, OCH_3_) or electron-withdrawing (e.g., Br, Cl, NO_2_) groups was introduced at the N-1 position of the tricyclic pyrazolobenzothiazine scaffold. All compounds were assessed for anti-HIV activity in PBMCs using the method of Schinazi [66], and cytotoxicity was evaluated on PBMC, CEM, and Vero cell lines according to Stuyver [67], with zidovudine (AZT) as the reference compound (Table 4).

Of the twenty-one compounds tested, only three (**10j**, **10l**, and **10m**) exhibited modest anti-HIV activity. The most active, compound **10m**, containing a 2,4-dihydroxyphenyl substituent, showed an EC_50_ of 3.3 μM. However, in comparison to the reference drug zidovudine (AZT, EC_50_ = 0.00037 μM), **10m** does not represent a promising candidate for further development. Moreover, **10m**, like **10j** and **10l**, exhibited significant cytotoxicity. These results indicate that the structural modifications introduced to the pyrazolobenzothiazine scaffold were not favorable for enhancing anti-HIV activity.

In 2015, Ahmad et al. reported the synthesis and anti-HIV activity of a new series of 1,2-benzothiazine derivatives [70]. In this series, a bulky substituent was introduced at the N-2 position of the pyrazolobenzothiazine scaffold. Fifteen compounds were obtained, divided into two series: **11** and **12**. In series **11**, the substituent was a benzylamide, whereas in series **12** it was an anilide (Table 5). As in previous studies, various electron-donating and electron-withdrawing groups were incorporated into the phenyl ring. All compounds were assessed for anti-HIV-1 activity in PBMCs using the method described previously [66], and cytotoxicity was evaluated in PBMC, CEM, and Vero cell lines, with zidovudine (AZT) serving as the reference compound (Table 5).

The study results indicated that anilide derivatives (series **12**) exhibited higher anti-HIV-1 activity than benzylamide derivatives (series **11**). In series **12**, all compounds except **12f** had EC_50_ values below 10 µM, with the highest activity observed for compounds bearing a bromine atom at the *ortho* or *meta* positions (**12c** and **12d**). Compound **12a** displayed similar activity, suggesting that the absence of a substituent at this position may also favorably influence antiviral activity. Among series **11**, only compound **11i** showed an EC_50_ below 10 µM. Nevertheless, the EC_50_ values of both series were considerably higher than that of the reference drug, AZT. Cytotoxicity studies revealed that most compounds in both series were cytotoxic, with series **12** derivatives showing the highest toxicity. Overall, structural modifications at the N-2 position of the pyrazolobenzothiazine scaffold enhanced anti-HIV activity compared to N-1-substituted derivatives; however, this increased activity was accompanied by greater cytotoxicity, indicating limited potential of these compounds as new anti-HIV lead structures.

In the same year, Khalid et al. reported the synthesis and evaluation of a new series of 1,2-benzothiazine derivatives [71]. Building on their 2013 work, the acethydrazide derivatives of pyrazolobenzothiazine were further modified to identify more potent HIV replication inhibitors [64]. In this series, heterocyclic rings in the acethydrazide moiety were replaced with phenyl substituents bearing electron-donating or electron-withdrawing groups (Table 6).

Fifteen compounds were obtained and assessed for anti-HIV-1 activity in PBMCs using the methodology applied in previous studies. Cytotoxicity was also evaluated in PBMC, CEM, and Vero cell lines, with zidovudine (AZT) serving as the reference compound in both assays. The study demonstrated that thirteen of the fifteen compounds exhibited anti-HIV activity, with EC_50_ values below 20 μM. The most active compound was **13c** (EC_50_ = 4.0 μM), bearing a *p*-chlorophenyl substituent, followed closely by **13i** (EC_50_ = 4.2 μM) with a 2,3,4-trimethoxyphenyl substituent, suggesting that the nature of the substituent had a limited impact on antiviral activity. However, the activity of all compounds was substantially lower than that of the reference drug, AZT. Cytotoxicity assays revealed that most compounds were highly toxic to all tested cell lines, with the exception of **13k**. A correlation between antiviral activity and cytotoxicity was observed, as increased activity often coincided with higher toxicity. These results indicate that further structural modifications are needed to improve the safety profile of this series of compounds.

In 2021, Imani et al. reported the synthesis and evaluation of new piroxicam derivatives, based on a 1,2-benzothiazine scaffold, as potential HIV integrase inhibitors [72]. Integrase (IN) is a viral enzyme responsible for inserting viral DNA into the host genome and is a critical component of the HIV life cycle, with no human homologue. Consequently, integrase inhibitors are expected to have minimal off-target effects [73,74,75,76]. Divalent Mg^2+^ ions play a key role in the enzyme’s function, and chelation of these ions by a ligand can inhibit the enzyme’s catalytic activity [77]. Piroxicam has been shown to chelate various metal ions, including Mn^2+^, Fe^2+^, Ni^2+^, Cu^2+^, and Zn^2+^, suggesting potential interactions with Mg^2+^ in the integrase active site [78,79,80,81]. Therefore, the 1,2-benzothiazine-3-carboxamide scaffold of piroxicam was considered a suitable platform for further optimization, as its hydroxyl and carboxamide groups form a chelating motif capable of coordinating Mg^2+^ ions. In the design of piroxicam analogs, a lipophilic substituent was moved from the C-3 position to the N-2 position to investigate the effect of this modification on anti-HIV activity (Table 7).

Fourteen derivatives, differing in the hydrophobic moiety at N-2, were synthesized and evaluated for inhibition of single-cycle HIV replication in HeLa cells. Cytotoxicity was also assessed in the same cell line. To gain insight into the mechanism of inhibition, molecular docking studies of the compounds with the HIV integrase protein were performed. Raltegravir served as a positive control, and piroxicam was included to assess the impact of the structural modifications [82].

The study demonstrated that most N-2-substituted derivatives (except **15i**) exhibited higher anti-HIV activity than compound **14**, which lacked a substituent at this position (EC_50_ = 105 μM). Similarly, compound **15b**, containing a cyclohexylmethyl group, showed weak activity (EC_50_ = 100 μM). The derivative with an aliphatic substituent, **15a**, was slightly more active than **14** and **15b** (EC_50_ = 80 μM) but less potent than the aromatic derivatives (**15c**–**m**, excluding **15i**). Compound **15i**, the only derivative bearing two chlorine atoms on the benzyl group, was the least active (EC_50_ = 110 μM), indicating that mono-substituted benzyl derivatives are more favorable than di-substituted ones (Table 7).

Among the aromatic derivatives, the most active compound was **15l** (EC_50_ = 20 μM), containing a *p*-fluorobenzyl substituent. Slightly lower activity was observed for **15d** and **15m** (EC_50_ = 25 μM), bearing *p*-methylbenzyl and *p*-nitrobenzyl substituents, respectively. Their anti-HIV activity was 5–6 times higher than that of piroxicam but more than 20 times lower than that of raltegravir. It was noted that *para*-substituted benzyl derivatives generally showed higher potency than *ortho*- or *meta*-substituted analogs.

Cytotoxicity studies revealed that all compounds, except **15b**, displayed relatively low toxicity (CC_50_ > 500 μM). Molecular docking studies further indicated that the derivatives interact with HIV integrase in a manner similar to co-crystallized raltegravir. These results suggest that additional structural modifications of the piroxicam scaffold are required to further enhance antiviral activity.

## 4. Structure–Activity Relationship (SAR) Analysis

The structure–activity relationship (SAR) analysis within the group of compounds targeting anti-HCV activity includes studies by Barreca et al. [56] and Manfroni et al. [63]. These teams investigated pyrazolobenzothiazine derivatives for their ability to inhibit NS5B polymerase, which plays a key role in HCV replication. Their results indicate that introducing a *p*-methanesulfonamidophenyl group and an amide bond at the C-3 position of the pyrazole ring increases the compounds’ affinity for NS5B polymerase. The position and nature of substituents on the N-1 phenyl ring also significantly influenced anti-NS5B activity. The most favorable substituents were fluorine, chlorine, and bromine, with fluorine showing the strongest effect. For fluorine substitution, activity increased in the order *meta < ortho < para*, whereas for chlorine or bromine derivatives, the trend was reversed: *ortho < para < meta*.

Cytotoxicity analysis showed that the *m*-fluoro derivative (compound **2a**) exhibited lower toxicity than the analogs derivatives **2b** and **2c**, which contained fluorine at the *ortho* or *para* positions, respectively. Subsequent studies demonstrated that the *p*-chloro derivative (compound **5b**) had a lower cytotoxic effect than the *m*-chloro derivative (**5a**). Moreover, chlorine-containing compounds were generally safer than their bromine analogs. Microscopic analysis aimed at evaluating subtle changes in cell morphology revealed that **5b** caused no significant morphological alterations while maintaining antiviral activity comparable to **2a**. These results suggest that *p*-chloro substitution on the phenyl ring is more favorable than *m*-fluoro substitution, both in terms of NS5B polymerase inhibition and the safety profile of the compounds. However, it should be noted that both derivatives exhibited substantially lower NS5B polymerase activity compared to the reference compound BTDZ, indicating the need for further structural optimization.

The structure–activity relationship (SAR) analysis within the group of compounds targeting anti-HIV activity includes studies by Aslam et al. [64], Ahmad et al. [70], Khalid et al. [71] and Imani et al. [72]. The research groups of Aslam, Ahmad, and Khalid synthesized and investigated four series of pyrazolobenzothiazine derivatives: (i) derivatives substituted at the thiazine nitrogen with acetohydrazide moieties containing a heterocyclic ring; (ii) derivatives substituted at the thiazine nitrogen with acetohydrazide moieties containing a phenyl substituent; (iii) derivatives substituted at the N-1 position of the pyrazole ring with acetohydrazide moieties containing a phenyl substituent; and (iv) derivatives substituted at the N-2 position of the pyrazole ring with benzylamide or anilide groups.

From the studies of all these compounds, a general conclusion can be drawn that, for anti-HIV activity, attaching a bulky substituent to the thiazine nitrogen is more favorable than substitution at the N-1 or N-2 positions of the pyrazole ring.

Among the compounds substituted at the N-1 position of the pyrazole, the most active was compound **10m**, which contained a phenyl ring with hydroxyl groups at positions 2 and 4 in the acylhydrazide moiety; however, it also exhibited cytotoxicity.

Compounds substituted at the N-2 position of the pyrazole with a benzylamide or anilide group showed higher anti-HIV activity than those substituted at N-1, although this was also associated with increased cytotoxicity. Anilide derivatives were slightly more potent than benzylamide derivatives, and the introduction of a bromine atom into the anilide ring further enhanced activity. The most active derivative in this series was compound **12d**, bearing a bromine atom at the *meta* position of the anilide moiety; however, it was also cytotoxic.

Among substituents at the thiazine nitrogen, heterocyclic rings (thiophene or thiazole) proved more advantageous than a phenyl substituent, as the latter increased compound cytotoxicity. Among the compounds containing a heterocyclic ring, the highest activity was observed for compound **9i**, bearing a thiophene ring substituted with two chlorine atoms, and compound **9h**, bearing a 2-amino-4-methylthiazole substituent. However, compound **9h** was non-cytotoxic, unlike compound **9i**, making it the most promising structure among all compounds studied. It should be emphasized that the anti-HIV activity of all compounds reported by these teams was significantly lower compared to the reference compound zidovudine. These results suggest that further structural modifications are required to improve both the potency and safety profile of these compounds as potential inhibitors of HIV replication.

Meanwhile, the team of Imani et al. was the only group to investigate the 1,2-benzothiazine scaffold not conjugated with a pyrazole ring. In the search for HIV integrase inhibitors, it was found that this scaffold represents a promising platform for structural optimization, as it is capable of chelating magnesium ions in the enzyme active site. The introduction of a lipophilic substituent at the thiazine nitrogen increased antiviral activity compared to the reference compound piroxicam; however, the activity remained lower than that of the reference inhibitor raltegravir.

Analysis of the influence of substituents on activity revealed that aromatic substituents were more favorable than aliphatic ones, and monosubstituted benzyl derivatives outperformed disubstituted analogs. The position of the substituent on the aromatic ring also had a significant impact on activity, with the highest activity observed for *para* substitution. The most active compound was the *p*-fluorobenzyl derivative **15l**, which also exhibited the highest selectivity index. Most of the synthesized compounds showed low cytotoxicity, and molecular docking studies confirmed that their binding mode to HIV integrase was similar to that of raltegravir. However, due to their significantly lower anti-HIV activity compared to the reference compound, further structural optimization is required.

## 5. Conclusions

In conclusion, although some 1,2-benzothiazine derivatives displayed measurable antiviral activity against HCV and HIV, their overall potency remained limited. Compound **5b** was the most active derivative against HCV, demonstrating inhibitory activity against NS5B polymerase (IC_50_ = 7.9 μM) without detectable cytotoxicity, whereas compound **9h** showed the highest anti-HIV activity (EC_50_ = 3.8 μM) in PBM cells, with no detectable cytotoxicity under the tested conditions. However, even the most active compounds against HCV and HIV exhibited substantially lower potency than the corresponding reference drugs. These findings indicate that, although the 1,2-benzothiazine scaffold may provide a basis for further investigation, substantial structural optimization will be required to achieve antiviral potency comparable to that of established reference compounds.

## Figures and Tables

**Table 1:** Results of anti-HCV and cytotoxicity studies of compounds **1**–**4**.

	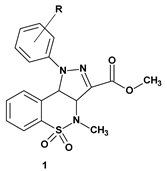			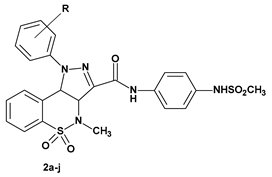			
	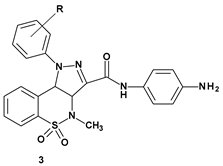			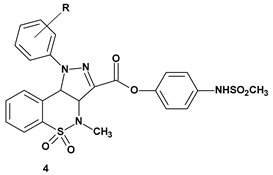			
**Compound**	**R**	**SPR on NS5B** **KD [μM]**	**Anti-NS5B Assay** **IC_50_ [μM]**	**Replicon Assay on Huh 5-2 Cells**			
				**EC_50_ [μM]**	**EC_90_ [μM]**	**CC_50_ [μM]**	**SI**
**1**	3-F	>300	ND	172 ± 10	ND	>323	2
**2a**	**3-F**	**75 ± 12**	**21.0 ± 2.8**	**7.5 ± 2.5**	**42 ± 2**	**>370**	**>49**
**2b**	2-F	162 ± 63	14.2 ± 0.6	6.4 ± 0.2	ND	>231	>36
**2c**	4-F	ND	3.9 ± 0.3	23 ± 13	ND	>231	>10
**2d**	H	53 ± 6	19.8 ± 3.6	14 ± 1	ND	>239	>17
**2e**	3-NO_2_	14 ± 6	ND	>220	>220	>220	≥1
**2f**	2-NO_2_	35 ± 3	20 ± 0.4	17.8 ± 0.1	ND	>220	>12.4
**2g**	4-NO_2_	ND	7.7 ± 1.3	7.9 ± 1.0	ND	55 ± 8	7.0
**2h**	3-NH_2_	163 ± 8	ND	9.2 ± 0.9	ND	20 ± 6	2.2
**2i**	2-NH_2_	106 ± 42	39.9 ± 1.7	26 ± 2	ND	>232	>9
**2j**	4-NH_2_	165 ± 16	18.2 ± 2.1	12 ± 1	ND	25 ± 5	2
**3**	3-F	161 ± 103	ND	19 ± 2	ND	>270	>14
**4**	3-F	63 ± 8	ND	88 ± 10	ND	>185	>2

ND = not determined.

**Table 2:** Results of anti-HCV and cytotoxicity studies of compounds **5**–**8**.

	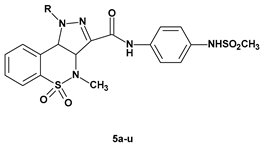		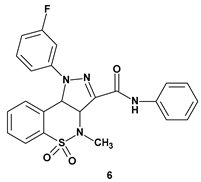			
	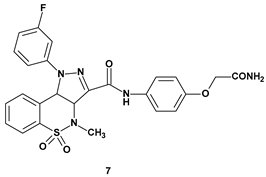		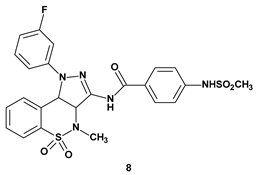			
**Compound**	**R**	**Anti-NS5B Assay IC_50_ [μM]**	**Cell-Based Replicon Assay**			
			**EC_50_ [μM]**	**EC_90_ [μM]**	**CC_50_ [μM]**	**SI**
**2a**	(3-F)phenyl	21.0 ± 2.8	7.5 ± 2.5	42 ± 2	>370	>49
**5a**	(3-Cl)phenyl	4.3 ± 0.2	9.8 ± 3.2	41 ± 11	>179	>18
**5b**	**(4-Cl)phenyl**	**7.9 ± 0.3**	**8.1 ± 4.3**	**23.3 ± 11.4**	**>224**	**>28**
**5c**	(2-Cl)phenyl	17.8 ± 0.5	7.4 ± 2.1	20.8 ± 3.9	>224	>30
**5d**	(3-Br)phenyl	7.7 ± 0.2	8.3 ± 0.5	26 ± 5	>166	>20
**5e**	(4-Br)phenyl	12.1 ± 0.5	7.4 ± 0.8	21 ± 5	>166	>22
**5f**	(2-Br)phenyl	26.5 ± 3.3	12.0 ± 0.4	ND	107 ± 34	8.9
**5g**	(3-CH_3_)phenyl	21.2 ± 4.5	5.1 ± 0.6	11.8 ± 1.2	>233	>46
**5h**	(4-CH_3_)phenyl	14.7 ± 0.2	4.8 ± 1.7	19.6 ± 11.8	>186	39
**5i**	(2-CH_3_)phenyl	30.8 ± 3.8	14.3 ± 1.1	ND	>233	>16
**5j**	(3-OCH_3_)phenyl	NA	11 ± 2	ND	>181	>16
**5k**	(4-OCH_3_)phenyl	NA	8.1 ± 1.5	25.0 ± 7.3	>226	>28
**5l**	(3-CF_3_)phenyl	23.7 ± 0.9	7.7 ± 1.9	33.2 ± 24.9	>211	>28
**5m**	(4-CF_3_)phenyl	36.3 ± 4.2	7.3 ± 0.8	31.2	>169	>23
**5n**	(2-CF_3_)phenyl	NA	25.0 ± 3	ND	>169	>7
**5o**	(3,4-diF)phenyl	25.4 ± 3.8	13.9 ± 0.4	ND	138 ± 24	9.9
**5p**	(3,4-diCl)phenyl	2.7 ± 0.3	9.7 ± 1.8	ND	>168	>17
**5q**	(3,4-diCH_3_)phenyl	NA	6.2 ± 0.3	20 ± 2	>181	>29
**5r**	(3-Cl,4-CH_3_)phenyl	8.0 ± 1.0	7.0 ± 2.0	37 ± 4	>175	>25
**5s**	(3-CF_3_,4-Cl)phenyl	NA	6.4 ± 1.7	ND	>160	>25
**5t**	cyclohexyl	43.0 ± 0.7	4.5 ± 0.6	11 ± 2	44 ± 38	9.7
**5u**	H	NA	88.2 ± 42.8	65.2	155 ± 50.5	2.5
**6**	-	NA	4.5 ± 0.6	ND	56	12
**7**	-	NA	15 ± 2	34 ± 7	>192	>13
**8**	-	NA	80.8 ± 9.4	ND	160 ± 18.2	2.3

NA = Not active; ND = not determined.

**Table 3:** Results of anti-HIV and cytotoxicity studies of compounds **9**.

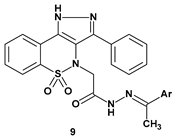						
**Compound**	**Ar**	**Anti-HIV-1 Activity in** **PBMCs [μM]**		**Cytotoxicity** **IC_50_ [μM]**		
		**EC_50_**	**EC_90_**	**PBM**	**CEM**	**Vero**
**9a**	Thiophen-2-yl	11.2	45.2	88.0	23.3	77.2
**9b**	3-Methylthiophen-2-yl	11.6	>100	56.2	13.1	75.3
**9c**	Thiophen-3-yl	24.3	89.3	73.2	10.7	78.7
**9d**	2,5-Dimethylthiophen-3-yl	>100	>100	36.5	17.6	44.4
**9e**	3-Chlorothiophen-2-yl	17.4	61.2	69.1	16.8	>100
**9f**	3-Bromothiophen-2-yl	>100	>100	42.8	19.6	>100
**9g**	5-Chlorothiophen-2-yl	>100	>100	13.9	8.4	57.4
**9h**	**2-amino-4-methylthiazole-5-yl**	**3.8**	**>100**	**>100**	**>100**	**>100**
**9i**	2,5-Dichlorothiophen-3-yl	3.2	38.8	12.2	14.4	38.8
**9j**	4-Methylthiophen-2-yl	13.3	>100	>100	18.3	6.0
**9k**	Coumarin-3-yl	22.9	85.6	82.7	27.8	>100
**9l**	Naphthalen-2-yl	>100	>100	12.2	24.2	<1
**9m**	5-Methylthiophen-2-yl	12.3	>100	62.3	13.8	30.6
**9n**	Benzo[b]furan-2-yl	79.0	>100	>100	22.4	23.0
**9o**	Pyrrole-2-yl	>100	>100	41.4	20.0	22.3
**9p**	5-Bromothiophen-2-yl	>100	>100	10.5	<1	18.1
**9q**	1,4-Benzodioxane-6-yl	>100	>100	67.1	23.9	>100
**9r**	5-Nitrothiophen-2-yl	>100	>100	85.0	5.0	43.6
**9s**	5-Methylfuran-2-yl	75.7	>100	53.8	15.4	>100
**9t**	2,5-Dimethylfuran-3-yl	>100	>100	17.3	9.9	41.4
**9u**	Thiazolo-2-yl	>100	>100	15.7	8.2	43.1
**9v**	Pyridino-4-yl	80.5	>100	75.6	34.5	84.1
**9w**	Thiophen-2-yl	70.2	>100	30.6	13.9	41.8
AZT	-	0.00037	0.014	>100	14.3	56.0

**Table 4:** Results of anti-HIV and cytotoxicity studies of compounds **10**.

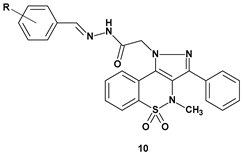						
**Compound**	**R**	**Anti-HIV-1 Activity in PBMCs [μM]**		**Cytotoxicity** **IC_50_ [μM]**		
		**EC_50_**	**EC_90_**	**PBM**	**CEM**	**Vero**
**10a**	4-Bromophenyl	>100	>100	97.0	46.9	36.4
**10b**	2,6-Dichlorophenyl	>100	>100	>100	78.6	>100
**10c**	4-Chlorophenyl	>100	>100	>100	44.9	20.5
**10d**	4-Dimethylaminophenyl	>100	>100	14.4	>100	>100
**10e**	2-Nitrophenyl	>100	>100	>100	88.9	45.8
**10f**	3-Nitrophenyl	>100	>100	>100	99.6	>100
**10g**	4-Diethylaminophenyl	>100	>100	>100	>100	23.5
**10h**	2,4-Dichlorophenyl	>100	>100	>100	>100	88.9
**10i**	2,3,4-Trimethoxyphenyl	>100	>100	>100	>100	19.6
**10j**	4-Methoxyphenyl	17.1	53.9	>100	20.7	15.4
**10k**	2,4-Dimethoxyphenyl	99.8	>100	>100	>100	>100
**10l**	3-Ethoxy-4-hydroxyphenyl	5.4	15.5	8.7	<1	9.2
**10m**	**2,4-Dihydroxyphenyl**	**3.3**	**5.9**	**7.8**	**<1**	**4.3**
**10n**	2-Fluorophenyl	>100	>100	>100	23.4	>100
**10o**	3,4,5-Trimethoxyphenyl	>100	>100	>100	75.4	>100
**10p**	2-Bromophenyl	>100	>100	>100	36.6	>100
**10q**	4-Acetamidophenyl	>100	>100	>100	44.6	>100
**10r**	3,4-Dichlorophenyl	>100	>100	38.0	19.3	>100
**10s**	4-Nitrophenyl	>100	>100	>100	>100	>100
**10t**	3-Chlorophenyl	>100	>100	51.6	12.1	78.5
**10u**	3-Bromophenyl	>100	>100	31.8	17.6	>100
AZT	-	0.00037	0.014	>100	14.3	56.0

**Table 5:** Results of anti-HIV and cytotoxicity studies of compounds **11** and **12**.

	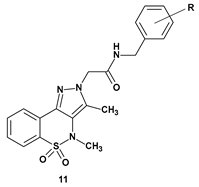		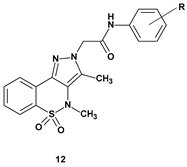			
**Compound**	**R**	**Anti-HIV-1 Activity in PBMCs [μM]**		**Cytotoxicity** **IC_50_ [μM]**		
		**EC_50_**	**EC_90_**	**PBM**	**CEM**	**Vero**
**11a**	2-Cl	15.9	28.5	74.2	68.4	38.7
**11b**	2-F	57.5	>100	87.5	82.0	96.1
**11c**	4-F	22.4	>100	>100	41.9	>100
**11d**	2-Br	16.7	87.5	63.1	38.1	36.5
**11e**	2-OCH_3_	17.2	60.3	>100	>100	>100
**11f**	3-OCH_3_	21.1	92.0	18.7	28.8	31.1
**11g**	4-OCH_3_	18.6	>100	>100	>100	73.0
**11h**	2-CH_3_	>100	-	>100	>100	>100
**11i**	4-CH_3_	9.9	77.7	52.2	>100	63.1
**12a**	H	6.8	16.3	7.7	<1.0	6.8
**12b**	2-F	7.6	35.4	18.0	9.5	28.2
**12c**	2-Br	6.5	15.7	2.4	6.0	8.2
**12d**	**3-Br**	**6.3**	**15.3**	**8.1**	**5.2**	**7.2**
**12e**	3-OCH_3_	7.2	17.3	10.5	9.3	>100
**12f**	2,4-(OCH_3_)_2_	>100	-	>100	>100	>100
AZT	-	0.003333	0.031	>100	14.3	56.0

**Table 6:** Results of anti-HIV and cytotoxicity studies of compounds **13**.

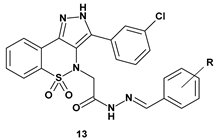						
**Compound**	**R**	**Anti-HIV-1 Activity in PBMCs [μM]**		**Cytotoxicity** **IC_50_ [μM]**		
		**EC_50_**	**EC_90_**	**PBM**	**CEM**	**Vero**
**13a**	4-F	9.2	26.7	20.0	31.6	45.7
**13b**	3-Cl	>10	>10	36.3	31.6	16.4
**13c**	**4-Cl**	**4.0**	**27.1**	**12.8**	**2.5**	**19.2**
**13d**	3-Br	>10	>10	22.9	12.9	>100
**13e**	4-Br	5.2	22.2	66.7	5.0	46.5
**13f**	2,4-diCl	19.6	76.3	80.7	13.8	84.8
**13g**	2,6-diCl	12.5	41.5	52.5	12.5	60.5
**13h**	3-OCH_3_	>10	>10	19.0	31.6	22.3
**13i**	2,3,4-triOCH_3_	4.2	23.8	21.8	31.6	29.0
**13j**	3,4,5-triOCH_3_	5.8	13.5	11.0	31.6	14.3
**13k**	2-NO_2_	>100	>100	>100	>100	>100
**13L**	3-NO_2_	92.4	>100	>100	47.3	27.0
**13m**	4-NO_2_	7.5	20.4	16.7	2.8	21.2
**13n**	4-COOH	8.3	23.0	17.2	13.4	18.2
**13o**	4-N(CH_3_)_2_	5.8	13.3	23.7	2.5	10.4
AZT	-	0.0033	0.031	>100	14.3	56.0

**Table 7:** Results of anti-HIV and cytotoxicity studies of compounds **14** and **15**.

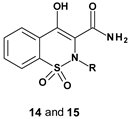				
**Compound**	**R**	**EC_50_ [μM]**	**CC_50_ [μM]**	**SI**
**14**	H	105	950	9.0
**15a**	ethyl	80	550	6.9
**15b**	cyclohexylmethyl	100	400	4.0
**15c**	benzyl	45	600	13.3
**15d**	4-methylbenzyl	25	680	27.2
**15e**	2-methylbenzyl	55	650	11.8
**15f**	3-methoxybenzyl	50	640	12.8
**15g**	2-chlorobenzyl	40	650	16.2
**15h**	4-chlorobenzyl	55	610	11.1
**15i**	3,5-dichlorobenzyl	110	510	4.6
**15j**	2-fluorobenzyl	35	680	19.4
**15k**	3-fluorobenzyl	60	550	9.2
**15l**	**4-fluorobenzyl**	**20**	**650**	**32.5**
**15m**	4-nitrobenzyl	25	650	26.0
piroxicam	-	120	900	7.5
raltegravir	-	0.9	>500	-

## Data Availability

No new data were created or analyzed in this study. Data sharing is not applicable to this article.

## Abbreviations

The following abbreviations are used in this manuscript:

AIDS	Acquired Immunodeficiency Syndrome
ART	Antiretroviral Therapy
AZT	Zidovudine
CC_50_	50% cytotoxic concentration
DAAs	Direct-Acting Anti-Hepatitis C Virus Drugs
EC_50_	50% effective concentration
EC_90_	90% effective concentration
FI	Fusion Inhibitors
HCV	Hepatitis C Virus
HIV	Human Immunodeficiency Virus
IC_50_	50% inhibitory concentration
IN	Integrase
InSTI	Integrase Strand Transfer Inhibitors
KD	Dissociation constant
NNRTI	Non-Nucleoside Reverse Transcriptase Inhibitors
NRTI	Nucleoside Reverse Transcriptase Inhibitors
NS5B	Nonstructural protein 5B
PBM	Peripheral Blood Mononuclear cells
PI	Protease Inhibitors
SI	Selectivity Index (ratio of CC_50_ to EC_50_)

## References

[1] Lozach P.-Y. (2020). Cell Biology of Viral Infections. Cells.

[2] Huerta L. (2020). Editorial: Anti-infective 2020: HIV—From pathogenesis to treatment. Curr. Opin. Pharmacol..

[3] Weng W.-K., Levy S. (2003). Hepatitis C Virus (HCV) and Lymphomagenesis. Leuk. Lymphoma.

[4] Weiss R.A. (1993). How does HIV cause AIDS?. Science.

[5] Stelzle D., Tanaka L.F., Lee K.K., Khalil A.I., Baussano I., Shah A.S.V., McAllister D.A., Gottlieb S.L., Klug S.J., Winkler A.S. (2020). Estimates of the global burden of cervical cancer associated with HIV. Lancet Glob. Health.

[6] Khandwala P., Singhal S., Desai D., Parsi M., Potdar R. (2021). HIV-Associated Anal Cancer. Cureus.

[7] Hentrich M., Pfister D. (2017). HIV-Associated Urogenital Malignancies. Oncol. Res. Treat..

[8] Dhokotera T., Bohlius J., Egger M., Spoerri A., Ncayiyana J.R., Naidu G., Olago V., Zwahlen M., Singh E., Muchengeti M. (2021). Cancer in HIV-positive and HIV-negative adolescents and young adults in South Africa: A cross-sectional study. BMJ Open.

[9] Rodger A.J., Cambiano V., Bruun T., Vernazza P., Collins S., Degen O., Corbelli G.M., Estrada V., Geretti A.M., Beloukas A. (2019). Risk of HIV transmission through condomless sex in serodifferent gay couples with the HIV-positive partner taking suppressive antiretroviral therapy (PARTNER): Final results of a multicentre, prospective, observational study. Lancet.

[10] Maheshwari A., Thuluvath P.J. (2010). Management of Acute Hepatitis C. Clin. Liver Dis..

[11] Strumillo S.T., Kartavykh D., de Carvalho F.F., Cruz N.C., de Souza Teodoro A.C., Diaz R.S., Curcio M.F. (2021). Host–virus interaction and viral evasion. Cell Biol. Int..

[12] Han J.J., Song H.A., Pierson S.L., Shen-Gunther J., Xia Q. (2023). Emerging Infectious Diseases Are Virulent Viruses—Are We Prepared? An Overview. Microorganisms.

[13] World Health Organization (2025). HIV and AIDS.

[14] World Health Organization (2025). Hepatitis C.

[15] Autran B., Carcelain G., Li T.S., Blanc C., Mathez D., Tubiana R., Katlama C., Debre P., Leibowitch J. (1997). Positive Effects of Combined Antiretroviral Therapy on CD4+ T Cell Homeostasis and Function in Advanced HIV Disease. Science.

[16] Lederman M.M., Connick E., Landay A., Kuritzkes D.R., Spritzler J., Clair M.S., Kotzin B.L., Fox L., Chiozzi M.H., Leonard J.M. (1998). Immunologic Responses Associated with 12 Weeks of Combination Antiretroviral Therapy Consisting of Zidovudine, Lamivudine, and Ritonavir: Results of AIDS Clinical Trials Group Protocol 315. J. Infect. Dis..

[17] Zolopa A.R., Andersen J., Komarow L., Sanne I., Sanchez A., Hogg E., Suckow C., Powderly W. (2009). Early Antiretroviral Therapy Reduces AIDS Progression/Death in Individuals with Acute Opportunistic Infections: A Multicenter Randomized Strategy Trial. PLoS ONE.

[18] Phanuphaka N., Gulick R.M. (2020). HIV treatment and prevention 2019: Current standards of care. Curr. Opin. HIV AIDS.

[19] Basit H., Koirala J., Hepatitis C. (2023). StatPearls.

[20] Gandhi R.T., Bedimo R., Hoy J.F., Landovitz R.J., Smith D.M., Eaton E.F., Lehmann C., Springer S.A., Sax P.E., Thompson M.A. (2023). Antiretroviral Drugs for Treatment and Prevention of HIV Infection in Adults: 2022 Recommendations of the International Antiviral Society–USA Panel. JAMA.

[21] Shin Y.H., Park C.M., Yoon C.-H. (2021). An Overview of Human Immunodeficiency Virus-1 Antiretroviral Drugs: General Principles and Current Status. Infect. Chemother..

[22] Micu S.I., Musat M., Dumitru A., Paduraru D.N., Rogoveanu A., Dumitriu A., Paunica S., Balalau C., Popoiag R.E. (2020). Hepatitis C virus: Host, environmental and viral factors promoting spontaneous clearance. J. Mind Med. Sci..

[23] Pol S., Thompson A.J., Collins M., Venier E., Cotte L., Laguno Centeno M., Mera J., Reiberger T., Burroughs M., Semizarov D.G. (2025). Effectiveness and safety of glecaprevir/pibrentasvir for 8 weeks in the treatment of patients with acute hepatitis C: A single-arm retrospective study. Hepatology.

[24] Falade-Nwulia O., Suarez-Cuervo C., Nelson D.R., Fried M.W., Segal J.B., Sulkowski M.S. (2017). Oral Direct-Acting Agent Therapy for Hepatitis C Virus Infection: A Systematic Review. Ann. Intern. Med..

[25] Bhattacharya D., Aronsohn A., Price J., Lo Re V., the American Association for the Study of Liver Diseases–Infectious Diseases Society of America HCV Guidance Panel (2023). Hepatitis C Guidance 2023 Update: American Association for the Study of Liver Diseases–Infectious Diseases Society of America Recommendations for Testing, Managing, and Treating Hepatitis C Virus Infection. Clin. Infect. Dis..

[26] Chang K.-C., Tung S.-Y., Wei K.-L., Shen C.-H., Hsieh Y.-Y., Chen W.-M., Chen Y.-H., Chen C.-H., Yen C.-W., Xu H.-W. (2021). Real-World Efficacy and Safety of Pangenotypic Direct-Acting Antivirals against Hepatitis C Virus Infection in Taiwan. Sci. Rep..

[27] Tomasiewicz K., Flisiak R., Jaroszewicz J., Małkowski P., Pawłowska M., Piekarska A., Simon K., Zarębska-Michaluk D. (2023). Recommendations of the Polish Group of Experts for HCV for the Treatment of Hepatitis C in 2023. Clin. Exp. Hepatol..

[28] Lampertico P., Carrión J.A., Curry M., Turnes J., Cornberg M., Negro F., Brown A., Persico M., Wick N., Porcalla A. (2020). Real-world effectiveness and safety of glecaprevir/pibrentasvir for the treatment of patients with chronic HCV infection: A meta-analysis. J. Hepatol..

[29] Kausar S., Khan F.S., Mujeeb Ur Rehman M.I., Akram M., Riaz M., Rasool G., Khan A.H., Saleem I., Shamim S., Malik A. (2021). A review: Mechanism of action of antiviral drugs. Int. J. Immunopathol. Pharmacol..

[30] Pawlotsky J.-M. (2023). Antivirals against hepatitis viruses: Basic mechanisms. Comprehensive Guide to Hepatitis Advances.

[31] Garau J., Wilson W., Wood M., Carle J. (1997). Fourth-generation cephalosporins: A review of in vitro activity, pharmacokinetics, pharmacodynamics and clinical utility. Clin. Microbiol. Infect..

[32] Rusu A., Lungu I.A. (2020). The new fifth-generation cephalosporins—A balance between safety and efficacy. Rom. J. Pharm. Pract..

[33] Dave D., Mittal I. (2024). A Comprehensive Review of Cephalosporin Antibiotics: Pharmacokinetic/Pharmacodynamic Considerations, Extended Infusion Strategies, and Clinical Outcomes. PEXACY Int. J. Pharm. Sci..

[34] Aljuaid A., Allahyani M., Alsaiari A.A., Almehmadi M., Alsharif A., Asif M. (2024). Green Synthetic Methods of Oxazine and Thiazine Scaffolds as Promising Medicinal Compounds: A Mini-review. Curr. Org. Synth..

[35] Sainsbury M., Katritzky A.R., Ree C.W. (1984). Oxazines, Thiazines and their Benzo Derivatives. Comprehensive Heterocyclic Chemistry.

[36] Sabatini S., Gosetto F., Serritella S., Manfroni G., Tabarrini O., Iraci N., Brincat J.P., Carosati E., Villarini M., Kaatz G.W. (2012). Pyrazolo [4,3-c][1,2]benzothiazines 5,5-Dioxide: A Promising New Class of Staphylococcus aureus NorA Efflux Pump Inhibitors. J. Med. Chem..

[37] Cernicchi G., Di Gregorio A., Felicetti T., Rampacci E., Casari G., Armeni T., Romaldi B., Zefaj E., Passamonti F., Massari S. (2025). NorA Efflux Pump Inhibitors: Expanding SAR Knowledge of Pyrazolo [4,3-c][1,2]benzothiazine 5,5-Dioxide Derivatives. Arch. Pharm..

[38] Dudek-Wicher R.K., Szczęsniak-Sięga B.M., Wiglusz R.J., Janczak J., Bartoszewicz M., Junka A.F. (2020). Evaluation of 1,2-Benzothiazine 1,1-Dioxide Derivatives In Vitro Activity towards Clinical-Relevant Microorganisms and Fibroblasts. Molecules.

[39] Kim S., Ramu R., Kwon S., Lee S.-H., Kim C., Kang S., Rhee S., Bae M., Ahn S., Ha D. (2010). Discovery of cyclicsulfonamide derivatives as 11β-hydroxysteroid dehydrogenase 1 inhibitors. Bioorg. Med. Chem. Lett..

[40] Kim S., Kwon S., Chu S., Lee J., Narsaiah B., Kim C., Kang S., Kang N., Rhee S., Bae M. (2011). Identification of Cyclicsulfonamide Derivatives with an Acetamide Group as 11β-Hydroxysteroid Dehydrogenase 1 Inhibitors. Chem. Pharm. Bull..

[41] Chen X., Zhang S., Yang Y., Hussain S., He M., Gui D., Ma B., Jing C., Qiao Z., Zhu C. (2011). 1,2-Benzothiazine 1,1-dioxide carboxylate derivatives as novel potent inhibitors of aldose reductase. Bioorg. Med. Chem..

[42] Park J.S., Rhee S.D., Kang N.S., Jung W.H., Kim H.Y., Kim J.H., Kang S.K., Cheon H.G., Ahn J.H., Kim K.Y. (2011). Anti-diabetic and anti-adipogenic effects of a novel selective 11β-hydroxysteroid-dehydrogenase type 1 inhibitor, 2-(3-benzoyl)-4-hydroxy-1,1-dioxo-2H-1,2-benzothiazine-2-yl-1-phenylethanone (KR-66344). Biochem. Pharmacol..

[43] Krzystek-Korpacka M., Szczęśniak-Sięga B., Szczuka I., Fortuna P., Zawadzki M., Kubiak A., Mierzchała-Pasierb M., Fleszar M.G., Lewandowski Ł., Serek P. (2020). L-Arginine/Nitric Oxide Pathway Is Altered in Colorectal Cancer and Can Be Modulated by Novel Derivatives from Oxicam Class of Non-Steroidal Anti-Inflammatory Drugs. Cancers.

[44] Szczuka I., Wierzbicki J., Serek P., Szczęśniak-Sięga B.M., Krzystek-Korpacka M. (2021). Heat Shock Proteins Hspa1 and Hsp90aa1 Are Upregulated in Colorectal Polyps and Can Be Targeted in Cancer Cells by Anti-Inflammatory Oxicams with Arylpiperazine Pharmacophore and Benzoyl Moiety Substitutions at Thiazine Ring. Biomolecules.

[45] Środa-Pomianek K., Michalak K., Palko-Łabuz A., Uryga A., Szczęśniak-Sięga B., Wesołowska O. (2019). Simvastatin Strongly Augments Proapoptotic, Anti-Inflammatory and Cytotoxic Activity of Oxicam Derivatives in Doxorubicin-Resistant Colon Cancer Cells. Anticancer Res..

[46] Bluke Z., Paass E., Sladek M., Abel U., Kauss V. (2016). Synthesis of 3,4-dihydro-2H-1,2-benzothiazine-3-carboxylic acid 1,1-dioxides and their evaluation as ligands for NMDA receptor glycine binding site. J. Enzym. Inhib. Med. Chem..

[47] Shan Y.Y., Zhang C.M., Tang L.Q., Liu Z.P., Bearss N.R., Sarver J.G., Luniwal A., Erhardt P.W. (2011). Syntheses of 2,3-Diarylated 2H-Benzo[e][1,2]Thiazine 1,1-Dioxides and Their 3,4-Dihydro Derivatives, and Assessment of Their Inhibitory Activity against MCF-7 Breast Cancer Cells. Med. Chem..

[48] Saddique F.A., Zaib S., Jalil S., Aslam S., Ahmad M., Sultan S., Naz H., Iqbal M., Iqbal J. (2018). Synthesis, monoamine oxidase inhibition activity and molecular docking studies of novel 4-hydroxy-N′-[benzylidene or 1-phenyl ethylidene] -2H/methyl/benzyl-1,2-benzothiazine-3-carbohydrazide 1,1-dioxides. Eur. J. Med. Chem..

[49] Saeed A., Ehsan S., Zia-ur-Rehman M., Marshall E.M., Loesgen S., Saleem A., Giovannuzzi S., Supuran C.T. (2025). Synthesis, characterization, antimicrobial, cytotoxic and carbonic anhydrase inhibition activities of multifunctional pyrazolo-1,2-benzothiazine acetamides. Beilstein J. Org. Chem..

[50] Ashraf A., Ejaz S.A., Rahman S.U., Siddiqui W.A., Arshad M.N., Lecka J., Sévigny J., Zayed M.E.M., Asiri A.M., Iqbal J. (2018). Hybrid compounds from chalcone and 1,2-benzothiazine pharmacophores as selective inhibitors of alkaline phosphatase isozymes. Eur. J. Med. Chem..

[51] Aslam S., Zaib S., Ahmad M., Gardiner J.M., Ahmad A., Hameed A., Furtmann N., Gütschow M., Bajorath J., Iqbal J. (2014). Novel structural hybrids of pyrazolobenzothiazines with benzimidazoles as cholinesterase inhibitors. Eur. J. Med. Chem..

[52] Bihovsky R., Tao M., Mallamo J.P., Wells G.J. (2004). 1,2-Benzothiazine 1,1-dioxide alpha-ketoamide analogues as potent calpain I inhibitors. Bioorg Med. Chem. Lett..

[53] Hina S., Zaib S., Uroos M., Zia-ur-Rehman M., Munir R., Riaz H., Syed Q., Abidi S.H.I. (2023). N-Arylacetamide derivatives of methyl 1,2-benzothiazine-3-carboxylate as potential drug candidates for urease inhibition. R. Soc. Open Sci..

[54] Wells G.J., Tao M., Josef K.A., Bihovsky R. (2001). 1,2-Benzothiazine 1,1-dioxide P_2_-P_3_ peptide mimetic aldehyde calpain I inhibitors. J. Med. Chem..

[55] Fatima M., Siddiqui W.A., Choudhary M.I., Ashraf A., Niaz S., Raza M.A., Alam S.M., Ashfaq M., Tahir M.N., Dahlous K.A. (2024). Synthesis of dimeric 1,2-benzothiazine 1,1-dioxide scaffolds: Molecular structures, Hirshfeld surface analysis, DFT and enzyme inhibition studies. RSC Adv..

[56] Barreca M.L., Manfroni G., Leyssen P., Winquist J., Kaushik-Basu N., Paeshuyse J., Krishnan R., Iraci N., Sabatini S., Tabarrini O. (2013). Structure-based discovery of pyrazolobenzothiazine derivatives as inhibitors of hepatitis C virus replication. J. Med. Chem..

[57] Behrens S.E., Tomei L., De Francesco R. (1996). Identification and properties of the RNA dependent RNA polymerase of hepatitis C virus. EMBO J..

[58] Wu J.Z., Hong Z. (2003). Targeting NS5B RNA-dependent RNA polymerase for anti-HCV chemotherapy. Curr. Drug Targets Infect. Disord..

[59] Mayhoub A.S. (2012). Hepatitis C RNA-dependent RNA polymerase inhibitors: A review of structure-activity and resistance relationships; different scaffolds and mutations. Bioorg. Med. Chem..

[60] Barreca M.L., Iraci N., Manfroni G., Cecchetti V. (2011). Allosteric inhibition of the hepatitis C virus NS5B polymerase: In silico strategies for drug discovery and development. Future Med. Chem..

[61] Das D., Hong J., Chen S.H., Wang G., Beigelman L., Seiwert S.D., Buckman B.O. (2011). Recent advances in drug discovery of benzothiadiazine and related analogs as HCV NS5B polymerase inhibitors. Bioorg. Med. Chem..

[62] Tedesco R., Shaw A.N., Bambal R., Chai D., Concha N.O., Darcy M.G., Dhanak D., Fitch D.M., Gates A., Gerhardt W.G. (2006). 3-(1,1-Dioxo-2H-(1,2,4)-benzothiadiazin-3-yl)-4-hydroxy-2(1H)-quinolinones, potent inhibitors of hepatitis C virus RNA-dependent RNA polymerase. J. Med. Chem..

[63] Manfroni G., Manvar D., Barreca M.L., Kaushik-Basu N., Leyssen P., Paeshuyse J., Cannalire R., Iraci N., Basu A., Chudaev M. (2014). New Pyrazolobenzothiazine Derivatives as Hepatitis C Virus NS5B Polymerase Palm Site I Inhibitors. J. Med. Chem..

[64] Aslam S., Ahmad M., Zia-ur-Rehman M., Montero C., Detorio M., Parvez M., Schinazi R.F. (2014). Synthesis and anti-HIV-1 screening of novel N′-(1-(aryl)ethylidene)-2-(5,5-dioxido-3-phenylbenzo[e]pyrazolo [4,3-c][1,2]thiazin-4(1H)-yl)acetohydrazides. Arch. Pharm. Res..

[65] Kumar S., Gupta S., Rani V., Sharma P. (2022). Pyrazole containing anti-HIV agents: An update. Med. Chem..

[66] Schinazi R.F., Sommadossi P.J., Saalmann V., Cannon D.L., Xie M.Y., Hart G.C., Smith J.A., Hahn E.F. (1990). Activities of 3′-azido-3′-deoxythymidine nucleotide dimers in primary lymphocytes infected with human immunodeficiency virus type 1. Antimicrob. Agents Chemother..

[67] Stuyver L.J., Lostia S., Adams M., Mathew J.S., Pai B.S., Grier J., Tharnish P.M., Choi Y., Chong Y., Choo H. (2002). Antiviral activities and cellular toxicities of modified 2′,3′-dideoxy-2′,3′-didehydrocytidine analogues. Antimicrob. Agents Chemother..

[68] Cihlar T., Ray A.S. (2010). Nucleoside and nucleotide HIV reverse transcriptase inhibitors: 25 years after zidovudine. Antivir. Res..

[69] Aslam S., Ahmad M., Athar M.M., Ashfaq U.A., Gardiner J.M., Montero C., Detorio M., Parvez M., Schinazi R.F. (2014). Synthesis, molecular docking and antiviral screening of novel N′-substituted benzylidene-2-(4-methyl-5,5-dioxido-3-phenylbenzo[e]pyrazolo[4,3-c][1,2]thiazin-1(4H)-yl)acetohydrazides. Med. Chem. Res..

[70] Ahmad M., Aslam S., Rizvi S.U.F., Muddassar M., Ashfaq U.A., Montero C., Ollinger O., Detorio M., Gardiner J.M., Schinazi R.F. (2015). Molecular docking and antiviral activity of N-substituted benzyl/phenyl-2-(3,4-dimethyl-5,5-dioxidopyrazolo[4,3-c][1,2]benzothiazin-2(4H)-yl)acetamides. Bioorg. Med. Chem. Lett..

[71] Khalid Z., Aslam S., Ahmad M., Munawar M.A., Montero C., Detorio M., Parvez M., Schinazi R.F. (2015). Anti-HIV activity of new pyrazolobenzothiazine 5,5-dioxide-based acetohydrazides. Med. Chem. Res..

[72] Imani A., Soleymani S., Vahabpour R., Hajimahdi Z., Zarghi A. (2021). Design, Synthesis, Docking Study and Biological Evaluation of 4-Hydroxy-2H-benzo[e][1,2]thiazine-3-carboxamide 1,1-dioxide Derivatives as Anti-HIV Agents. Iran. J. Pharm. Res..

[73] Goldgur Y., Craigie R., Cohen G.H., Fujiwara T., Yoshinaga T., Fujishita T., Sugimoto H., Endo T., Murai H., Davies D.R. (1999). Structure of the HIV-1 integrase catalytic domain complexed with an inhibitor: A platform for antiviral drug design. Proc. Natl. Acad. Sci. USA.

[74] Kawasuji T., Yoshinaga T., Sato A., Yodo M., Fujiwara T., Kiyama R. (2006). A platform for designing HIV integrase inhibitors. Part 1: 2-hydroxy-3-heteroaryl acrylic acid derivatives as novel HIV integrase inhibitor and modeling of hydrophilic and hydrophobic pharmacophores. Bioorg. Med. Chem..

[75] Narang B.K., Grewal G.K., Roy S., Bariwal J., Gupta M.K., Rawal R.K. (2014). A novel integrase targeting agent to explore the future prospective of HIV eradication: Dolutegravir. Curr. HIV Res..

[76] Hazuda D.J. (2012). HIV integrase as a target for antiretroviral therapy. Curr. Opin. HIV AIDS.

[77] Mouscadet J.-F., Delelis O., Marcelin A.-G., Tchertanov L. (2010). Resistance to HIV-1 integrase inhibitors: A structural perspective. Drug Resist. Updat..

[78] Christofis P., Katsarou M., Papakyriakou A., Sanakis Y., Katsaros N., Psomas G. (2005). Mononuclear metal complexes with piroxicam: Synthesis, structure and biological activity. J. Inorg. Biochem..

[79] Cini R. (2000). Anti-inflammatory compounds as ligands in metal complexes as revealed in X-ray structural studies. Comments Inorg. Chem..

[80] Cini R., Giorgi G., Cinquantini A., Rossi C., Sabatini M. (1990). Metal complexes of the antiinflammatory drug piroxicam. Inorg. Chem..

[81] Hosseini S.M., Imani A., Rahimzadegan M., Mohammadi S., Golaghaei A. (2019). Synthesis and biological evaluation of Piroxicam derivative as a lead chelator. Main. Group Met. Chem..

[82] Hicks C., Gulick R.M. (2009). Raltegravir: The first HIV type 1 integrase inhibitor. Clin. Infect. Dis..

